# Cation and Spin Interactions in Cadmium Ferrite: A Quantum Mechanical Study

**DOI:** 10.3390/ijms26104912

**Published:** 2025-05-20

**Authors:** Tahani Saad Almutairi

**Affiliations:** Section of Physical Chemistry, Department of Chemistry, Taibah University, Madinah 42353, Saudi Arabia; talmutairi@taibahu.edu.sa

**Keywords:** spinel, spin, ferrite, magnetism, Raman

## Abstract

Spinel ferrites have emerged as fascinating materials, not just for their diverse functionalities, but for the dynamic structural transformations they undergo under varying conditions. These phase transitions, often subtle yet deeply influential, play a pivotal role in tuning their electronic, magnetic, and vibrational properties. At the heart of this complexity lies the versatile arrangement of divalent and trivalent cations between the tetrahedral (A) and octahedral (B) sites, giving rise to a rich spectrum of magnetic interactions, charge dynamics, and lattice responses. This intricate cation interplay makes spinel ferrites a playground for exploring structure–property relationships in advanced functional materials. In this study, we investigated the structural, vibrational, and magnetic properties of Cd ferrite using advanced hybrid functionals (B3LYP, HSE06, and PBE0). Our calculations reveal that the normal spinel phase is the most stable configuration, with minimal energy differences between spin arrangements (~0.005–0.008 eV) and slightly larger differences when including zero-point energy (~0.023 eV). All the investigated structures exhibit a semiconducting nature, with band gaps varying depending on the spin arrangements. The IR and Raman spectra highlight the influence of spin ordering on vibrational modes. The simulations of the Raman spectra demonstrate that both the frequencies and intensities of the Raman peaks strongly depend on the magnetic ordering. The present theoretical study offers a consistent framework for assigning vibrational modes, which may help resolve ambiguities and contribute to a deeper understanding of the vibrational properties of Cd ferrite. These findings provide a robust foundation for further experimental and computational exploration of this material.

## 1. Introduction

Cd ferrite stands as a significant member of the spinel ferrite family, characterized by its unique magnetic and electrical properties. These materials, represented by the general formula CdFe_2_O_4_, are part of a broader class of ferrites that play a pivotal role in modern technology due to their versatile applications in electronics, magnetism, and catalysis [1,2,3,4,5].

Cd ferrite crystallizes in a spinel structure, which is a specific type of cubic crystal structure. This structure is significant as it influences the magnetic properties of the material. In the spinel lattice, cadmium ions typically occupy tetrahedral (A) sites while the ferric ions Fe^3+^ reside in octahedral (B) sites [6,7]. The distribution of these ions in the lattice plays a crucial role in determining the magnetic nature of the ferrite. Ferrimagnetism in spinel arises from the antiparallel alignment of magnetic moments between different sublattices, which in the case of Cd ferrite involves the magnetic moments of Fe^3+^ ions at the octahedral sites. Since Cd^2+^ ions are non-magnetic, the net magnetization is primarily due to the unopposed magnetic moments of Fe^3+^ ions. The super-exchange interactions between Fe^3+^ ions through shared oxygen ions lead to a strong antiferromagnetic coupling. However, the overall structure results in a net magnetic moment in some cases, due to the unequal moments of the two sublattices [8]. The spin ordering and resultant magnetic properties of Cd ferrite are also temperature dependent [6,8,9]. At high temperatures, thermal agitation can overcome the magnetic ordering, leading to a transition. Imperfections in the crystal structure, such as cation inversion where some Cd^2+^ ions might occupy octahedral sites and some F^3+^ ions might occupy tetrahedral sites, can alter the magnetic interactions and thus the spin ordering [6,8,10,11,12,13].

The magnetic properties of Cd ferrite are essential for its use in magnetic recording media and electromagnetic devices [14]. Additionally, it is utilized in information storage, microwave absorption, magnetic cores, and magnetic fluids [15]. Furthermore, Cd ferrite serves as a catalyst in the degradation of organic pollutants and other chemical reactions [16]. It is also employed in the manufacture of gas sensors and biosensors, leveraging its sensitive electrical response to changes in the environment [12,17].

Identifying the structure of Cd ferrites using Raman spectroscopy presents several technical challenges, primarily due to the complexity of the spinel structure and the similarity of vibrational modes shared among various spinel ferrites [18,19]. These techniques are essential in characterizing the vibrational properties of materials, which in turn provide insights into their structural and chemical behavior. In Raman spectra, peaks corresponding to similar vibrational modes can overlap, especially in materials with multiple types of metal–oxygen bonds [20,21]. This overlap makes it difficult to assign specific peaks to particular vibrational modes without ambiguity. Peak splitting can also occur due to factors like crystal field effects, Jahn–Teller distortions, or lower symmetry in the crystal structure [22]. If there is any deviation from the ideal symmetry of the spinel structure (like distortion due to the size ions, it might cause splitting of some vibrational peaks [23,24,25]. Therefore, in present research, we investigated the effects of inversion and spin configurations on the Raman frequencies of Cd ferrite (X = 0.0, 0.5, 1.0) using density functional theory and hybrid functionals. We meticulously examined all possible cation distributions within tetrahedral and octahedral sites. This thorough analysis encompassed evaluations of relative energy, charge, and magnetic distributions, electronic structure, and vibrational frequencies, providing a comprehensive insight into the material’s properties.

## 2. Result and Discussion

### 2.1. Energy, Geometric Structure, Charge and Spin Distribution, and Electronic Structure

CdFO has a normal spinel structure and exhibits ferromagnetic behavior, with magnetization increasing as particle size decreases [8,24]. Table 1 provide insights into the energy differences among different degrees of inversion (X = 0, 0.5, and 1) and various spin arrangements—ferromagnetic (FM), ferrimagnetic (FRI), and antiferromagnetic (AFM)—of CdFO which were obtained using three functionals—B3LYP, HSE06, and PBE0. The degree of inversion (X) represents the fraction of Fe atoms that occupy tetrahedral (T_d_) sites in the spinel structure, influencing the material’s magnetic and structural properties. Table 1 presents the total energy (*E*) and relative energy (*E*ᵣ), showing that AFM configuration within X = 0.0 generally has the lowest relative energy, suggesting a preference for antiferromagnetic ordering in the normal spinel geometry. AFM is approximately 0.008 eV lower than that of ferromagnetic (FM) in normal geometry, with this energy gap expanding to 0.023 eV when considering free energy, inclusive of zero-point energy (Table 2). This suggests that temperature could significantly influence the stability of CdFO [8,26]. These energetic advantages diminish with increased inversion, while FM and FRI configurations become comparatively less stable across the three functionals. Moreover, Cd atoms show a preference for occupying the T_d_ site, which facilitates greater relaxation—similar to the behavior observed in Zn atoms within ZnFe_2_O_4_ [27]. However, this effect becomes more pronounced in CdFO, further stabilizing its structure.

**Figure 1:**
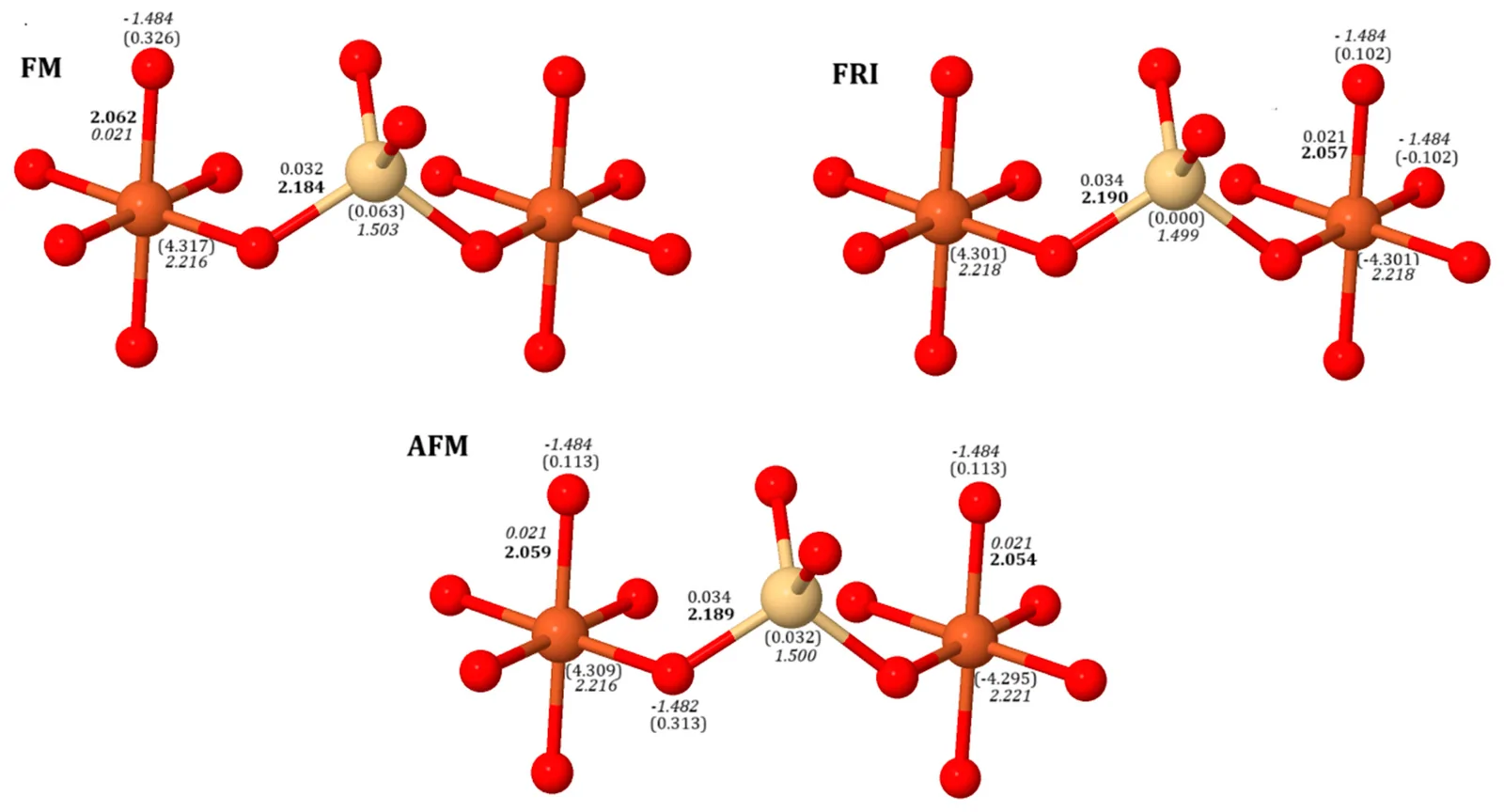
Structural and electronic parameters of normal spinel of CdFO, including bond lengths (Å, **bold**), bond populations (|e|), spin moments (|e|, in brackets), and Mulliken charges (|e|, *italicized*), using B3LYP. Color coding: oxygen (red), iron (orange), cadmium (yellow). In the ferromagnetic configuration (FM), the spin arrangement [↑ (α), ↓ (β)] in the primitive formula is (Co_2_)[ ↑Fe_4_]O_8_. For the ferrimagnetic (FRI) case, it is (Co_2_) [↑Fe_3_↓Fe], and for the antiferromagnetic (AFM) configuration, it is (Co_2_) [↑Fe_2_↓Fe_2_].

In the FM configuration, CdFO shows aligned spins, contributing to a net magnetization of about 10 μB per formula unit, with bond lengths around 2.184 Å for Cd-O and 2.062 Å for Fe-O interactions and relatively low bond population values, indicating a greater tendency towards ionic bonding nature. The spin moments are aligned, while the Mulliken charges reflect electron density primarily on oxygen atoms (1.48 |e|). In the AFM configuration, adjacent spins are oppositely aligned, resulting in magnetic moment cancellation. The bond lengths, such as the 2.190 Å Cd-O bond, are slightly adjusted from FM, reflecting minor lattice relaxation, and the Mulliken charges exhibit small shifts, showing subtle changes in charge distribution due to the opposite spin alignment. In the FRI configuration, partial spin cancellation leads to 5 μB net magnetic moment per formula characteristic of ferrimagnetic ordering, with Cd-O bond lengths around 2.189 Å and similar bond populations. Across all configurations, slight variations in bond lengths and Mulliken charges indicate how spin arrangements subtly influence CdFO’s geometry and electronic structure. This analysis suggests that the different magnetic arrangements in CdFO affect its bond characteristics, charge distribution, and overall structural stability. The visualization in Figure 1 provides insight into the fine structural changes induced by different spin arrangements within the normal geometry, helping to elucidate their impact on the electronic structure and stability.

Based on the data provided in Table 3, the magnetic moments of Fe, Cd, and O atoms in CdFO exhibit distinct values across various spin configurations and functional methods. For Fe atoms occupying octahedral (Oh) and tetrahedral (Td) sites, a consistent trend is observed, where FM arrangements generally yield higher magnetic moments compared to FRI and AFM configurations, regardless of the functional used. Specifically, Fe(Oh) moments remain in the range of 4.3–4.4 μB, reflecting high-spin Fe^3+^ behavior. The Td Fe sites, when occupied, exhibit similarly strong but oppositely signed magnetic moments due to antiparallel alignment in FRI and AFM cases.

For the Cd atoms, the magnetic moments are generally close to zero, indicating minimal magnetic contribution, which is expected given Cd’s typical non-magnetic nature in such ferrite structures. However, slight variations in the range of 0.01–0.07 μB can be observed across all configurations, which could be attributed to polarization effects in the surrounding environment.

Interestingly, oxygen atoms show non-negligible magnetic moments, especially in FM and FRI states, with values ranging from ~0.1 to 0.36 μB, depending on the configuration and functional. The observed magnetic moments on oxygen atoms originate from spin polarization induced by hybridization between Fe 3d and O 2p orbitals. While oxygen is inherently non-magnetic, the asymmetric spin distribution in spin-polarized configurations leads to a net moment on oxygen atoms. This phenomenon is a hallmark of super-exchange interactions, wherein magnetic coupling between transition metal cations is mediated through an intervening anion—in this case, oxygen.

In normal spinel CdFe_2_O_4_, all Fe^3+^ ions occupy octahedral (B) sites, and the dominant magnetic exchange occurs via Fe–O–Fe super-exchange paths. The geometry of these paths is critical: the Fe–O–Fe bond angles in a normal spinel are close to 90°, resulting from edge-sharing octahedra. According to the Goodenough–Kanamori–Anderson (GKA) rules [28], the nature and strength of super-exchange depend on (i) the angle of the M–O–M bond, (ii) the electronic configuration of the magnetic cations, and (iii) the overlap symmetry of the interacting orbitals.

For Fe^3+^ ions in high-spin 3d^5^ configuration (half-filled shells), the GKA rules state the following: A 180° Fe–O–Fe angle promotes strong antiferromagnetic coupling, due to maximal overlap of half-filled orbitals via oxygen p orbitals (σ-type overlap). A 90° Fe–O–Fe angle, by contrast, leads to weaker and often ferromagnetic or frustrated interactions, due to reduced orbital overlap (π-type or orthogonal overlap), and the symmetry of t_2_g orbitals.

Thus, in CdFe_2_O_4_, the close-to-orthogonal Fe–O–Fe angles reduce the strength of antiferromagnetic coupling, which explains the small static energy difference (~0.008 eV) between FM and AFM states. However, the AFM state remains energetically favored even at the static level, suggesting that weak antiferromagnetic coupling still dominates despite geometric limitations. This could be attributed to a residual overlap of t_2g_–p–t_2g_ orbitals via π-interactions or multiple exchange paths with small additive contributions. Notably, the B3LYP functional tends to enhance the spin polarization on oxygen more than others, likely due to its treatment of exact exchange, which amplifies localization and magnetic contrast.

**Figure 2:**
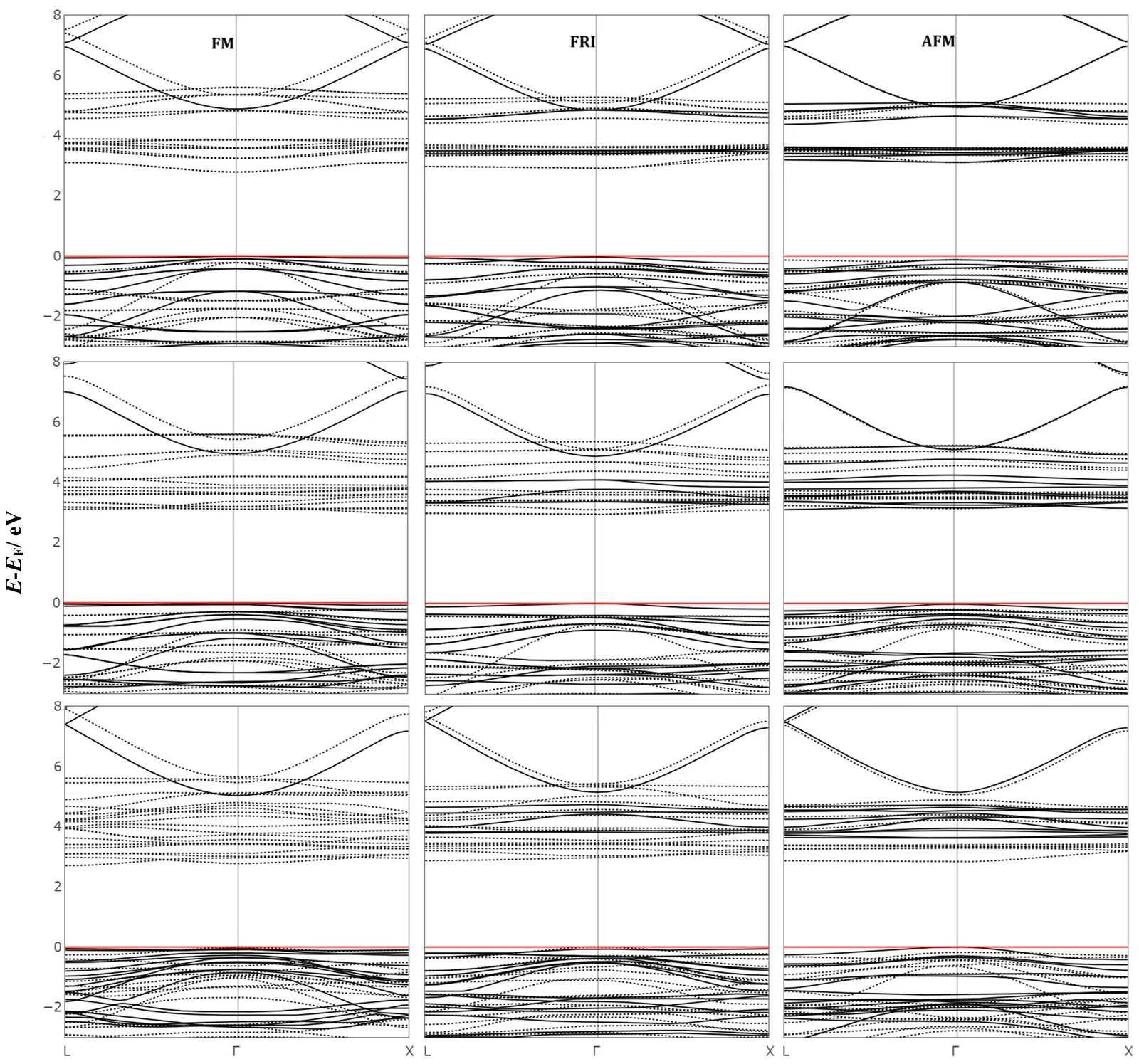
Calculated electronic band dispersion for CdFO in FM, FRI, and AFM phases. The panels, arranged from top to bottom, correspond to X = 0.0, X = 0.5, and X = 1.0, respectively. Calculations were performed using B3LYP, with solid lines representing spin-up states and dashed lines representing spin-down states. The Fermi level or VBM is set to zero for reference.

The gaps extracted from band dispersion curves (Figure 2) for CdFO are presented in Table 4 and reveal distinct electronic properties under various spin configurations and inversion degrees, calculated using different functionals. The spin-resolved electronic band gaps for the majority (α) and minority (β) spin channels, respectively, demonstrate significant variation depending on the spin arrangement, inversion degree, and the functional applied.

For X = 0.0, the FM configuration yields the highest band gaps across all functionals, with B3LYP predicting a direct gap (α) 4.884 and (β) 3.012 eV, while HSE06 and PBE0 provide comparable values, with PBE0 yielding the largest gaps. The FRI and AFM configurations produce lower band gaps, with the AFM state showing a particularly notable reduction, as B3LYP predicts a direct gap 3.112 eV for both spin channels. This reduction can be attributed to the partial cancellation of magnetic moments, which decreases the energy separation between the conduction and valence bands.

As the inversion degree increases to X = 0.5, the FM state again yields the largest band gap, with PBE0 predicting (α) 5.561 eV and (β) 3.392 eV. In the FRI configuration, the band gap along the majority spin channel decreases by approximately 0.2–0.3 eV compared to X = 0.0, with a corresponding increase in the minority gap. The AFM configuration at this inversion degree also demonstrates slightly reduced values compared to FM.

At X = 1.0, the FM configuration maintains the trend of larger band gaps, with B3LYP yielding (α) 5.078 eV. This behavior is likely due to full spin alignment, which minimizes electron–electron interactions and enhances spin polarization effects. In contrast, the FRI and AFM configurations show lower band gaps across all functionals, particularly along the majority spin channel.

Across all configurations and inversion degrees, the choice of functional significantly impacts the calculated band gaps. PBE0 consistently predicts the highest band gaps, followed by HSE06 and then B3LYP. This ordering reflects the differences in hybrid functional formulations, with PBE0’s higher Hartree–Fock exchange component enhancing band gap predictions. In summary, the data illustrate how spin arrangement, inversion degree, and functional choice collectively influence the electronic band structure of CdFO. While FM states consistently yield the largest band gaps, the effect of inversion degree on the band gap depends on the specific spin arrangement, functional used, and spin channel considered, highlighting the complex interplay of structural and magnetic factors in CdFO’s electronic properties.

### 2.2. Lattice Dynamics

**Figure 3:**
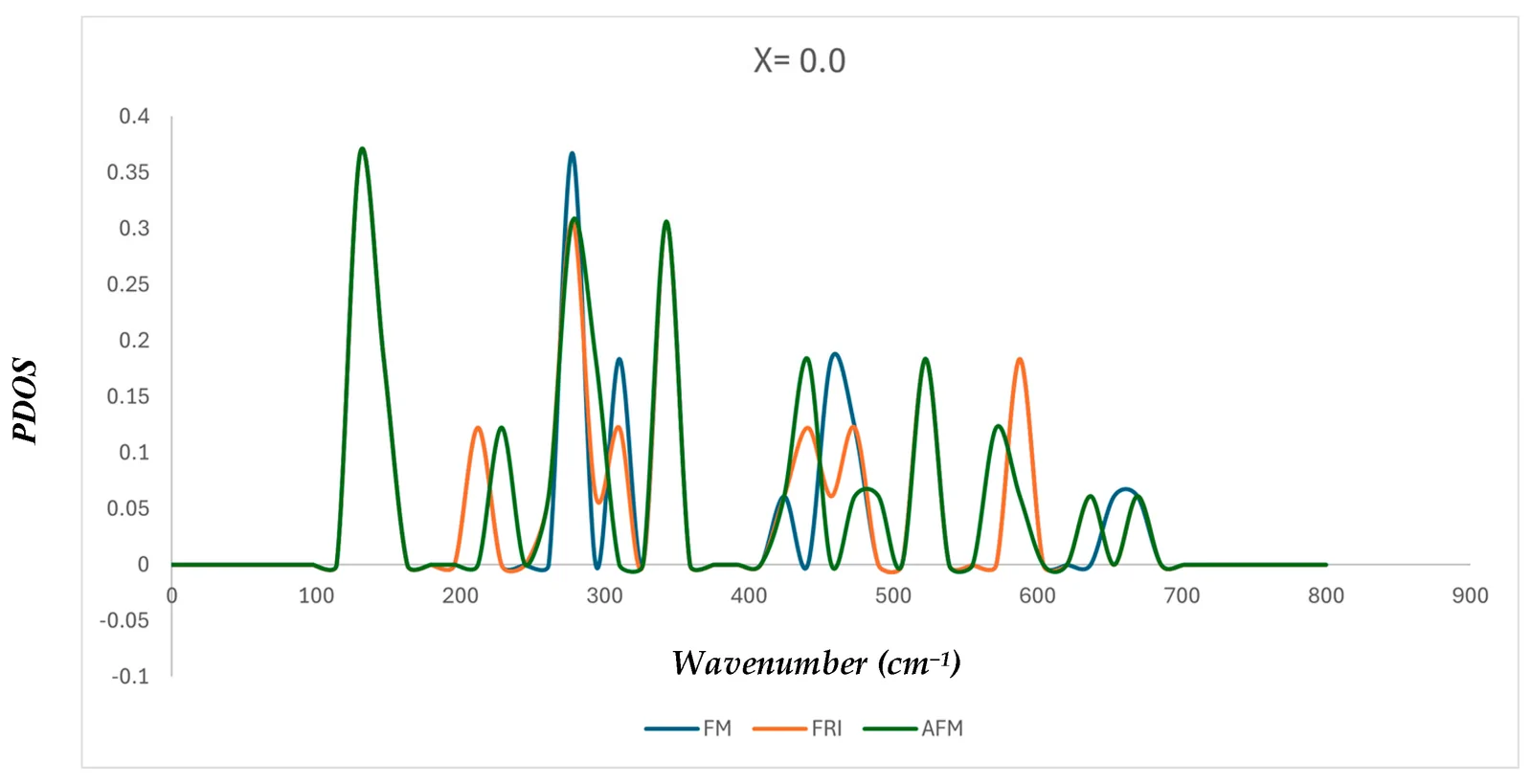
Calculated phonon density of states for CdFO in the normal spinel phase using the B3LYP functional.

To examine the influence of magnetic ordering on the vibrational properties of CdFe_2_O_4_, the phonon density of states (PDOS) was computed for the normal spinel structure (X = 0.0) in three distinct magnetic configurations: ferromagnetic (FM), ferrimagnetic (FRI), and antiferromagnetic (AFM). As shown in Figure 3, all three spin states exhibit vibrational activity across the ~100–700 cm^−1^ range, consistent with the typical phonon spectrum of spinel ferrites.

While the overall phonon band widths remain similar, noticeable differences are observed in the peak positions and intensities among the three configurations. For example, the FM state exhibits strong peaks around 300 and 500 cm^−1^, while the AFM and FRI configurations show slight shifts and redistributions of spectral weight in these regions. These variations suggest that the magnetic configuration influences the interatomic force constants, leading to minor shifts in phonon frequencies. Such sensitivity is characteristic of spin–phonon coupling, where the magnetic order alters the lattice dynamics through the modification of bonding interactions, particularly along Fe–O–Fe super exchange pathways. Importantly, no imaginary (negative) frequencies are observed in any configuration, confirming the dynamical stability of the normal spinel phase under FM, FRI, and AFM magnetic orderings. The observed vibrational features, especially the sharp peaks in the 250–600 cm^−1^ range, align well with Raman-active modes expected for spinel ferrites.

### 2.3. Spectroscopic Characterization

**Figure 4:**
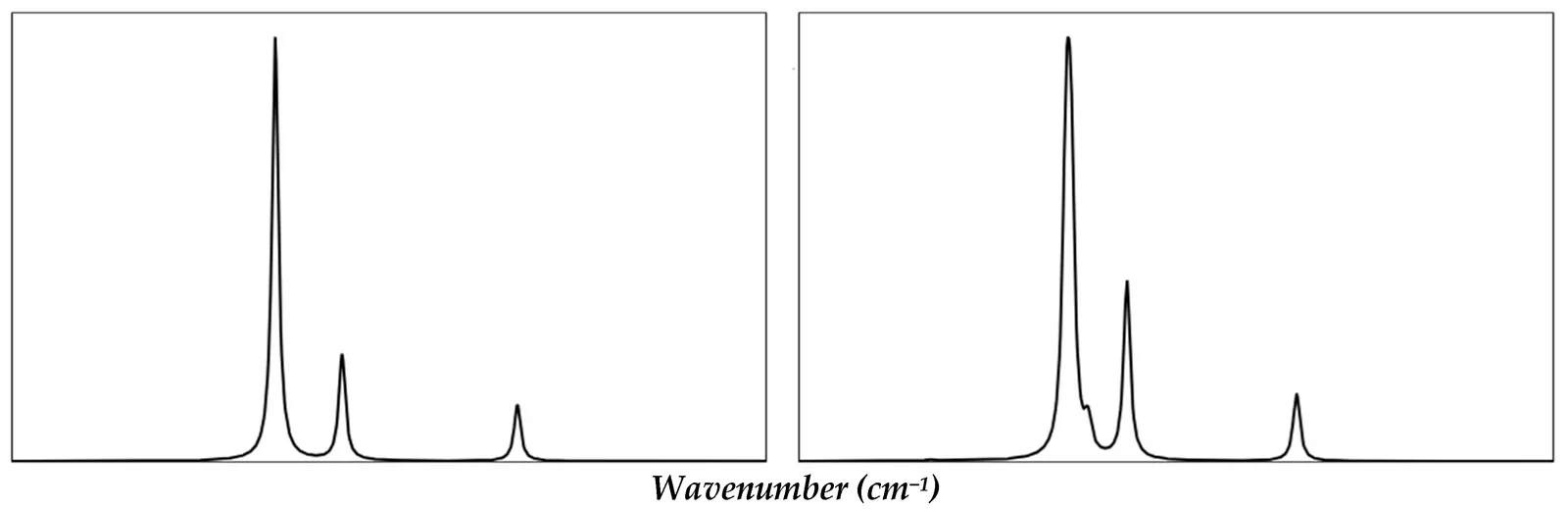
B3LYP-simulated infrared spectra of CdFO (X = 0.0) depicting two magnetic configurations; left panels for FM and right panels for AFM.

Raman and infrared (IR) spectroscopy are powerful tools for revealing the complex structures of crystals. However, experimental results can be affected by various factors such as preparation methods, temperature, and the presence of structural defects. To circumvent these challenges, simulated spectra serve as a reliable alternative, providing precise characterizations with reduced interference from these variables. The IR vibrational spectra of the most stable configurations of CdFO, denoted as X = 0.0, were calculated using the B3LYP level of theory and based on the equilibrium lattice structures. These results are illustrated in Figure 4. Within the Fd3m (Oh_7_) space group of the spinel structure, 42 vibrational modes were identified. This includes 39 optical modes and 3 acoustic modes [29]. Notably, all the 4T_1u_ modes predicted by group theory were observed in this study and are documented in Table 5 for comparison with existing literature data. The two T_1u_ modes in the lower frequency region are seldom detected experimentally. This is often due to limitations in the instrumentation and the presence of local defects and stress, which typically result in peak broadening [30]. Such broadening leads to the merging of the peaks at 279 cm^−1^ and 350 cm^−1^ into a single broad peak, which most studies assign to the *v*_2_ mode. However, our predicted frequency for the *v*_1_ mode aligns closely with experimental values, as listed in Table 5.

The vibrational Raman spectra of CdFO display distinctive patterns that reflect the material’s structural and bonding characteristics, while also highlighting deviations from the expected behavior of an ideal cubic spinel ferrite structure. In a perfect cubic spinel, group theory predicts five Raman-active modes (A_1g_, E_g_, and three T_2g_) resulting from symmetric vibrations of the cations and anions located in the lattice’s tetrahedral and octahedral sites. However, observations of additional or shifted modes and variations in intensity suggest the presence of structural distortions, cation inversion, or other factors affecting the vibrational properties. These variations highlight the intricate relationship between crystal symmetry, magnetic ordering, and electronic structure, which together influence the material’s vibrational characteristics and unique optical behavior.

**Figure 5:**
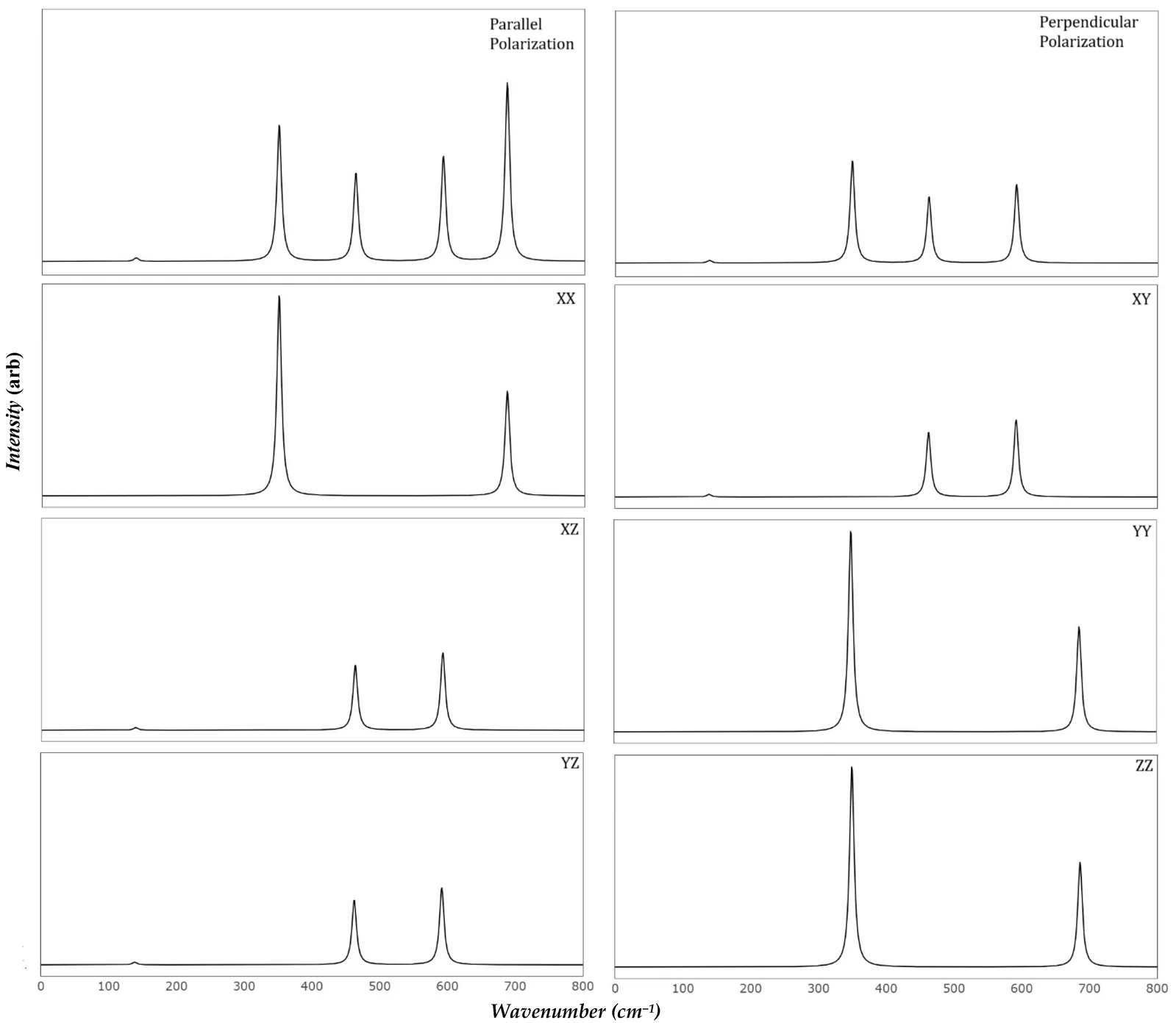
Polarized Raman spectra of CdFO (X = 0), illustrating the dependence of mode intensities on the excitation light direction. The top two panels represent the polycrystalline spectra, while the bottom panels show single-crystal spectra with polarization along different crystallographic directions.

The Raman spectra of CdFO reveals distinct anisotropic behavior, as evident from the comparison between polycrystalline and single-crystal results (Figure 5). In single-crystal spectra, strict Raman selection rules are followed, with the A_1g_ mode appearing only in parallel polarization (XX, YY, ZZ) and absent in perpendicular polarization (XY, XZ, YZ), while T_2g_ modes are exclusively observed in crossed polarization configurations. The absence of A_1g_ in perpendicular polarization arises from its symmetry properties, which only allow it to be Raman active when the incident and scattered light share the same polarization direction. Its strong intensity in parallel polarization further confirms this selection rule. In contrast, polycrystalline spectra exhibit minor selection rule relaxation, with T_2g_ modes appearing in both parallel and perpendicular polarizations due to random grain orientations. This anisotropic behavior highlights the role of crystallographic orientation in determining Raman mode visibility, emphasizing the necessity of polarization-resolved Raman analysis to accurately characterize phonon interactions in CdFO. Additionally, the Raman signal is inherently dependent on the incident light direction, which influences the relative intensity of observed peaks. While fundamental Raman selection rules remain unchanged, variations in incident light direction modify the scattering efficiency and peak intensities due to changes in light–matter interaction geometry. In the following sections, we will focus on presenting only the total intensity for the studied configurations to provide a clearer comparison of vibrational trends across different compositions and magnetic states

**Figure 6:**
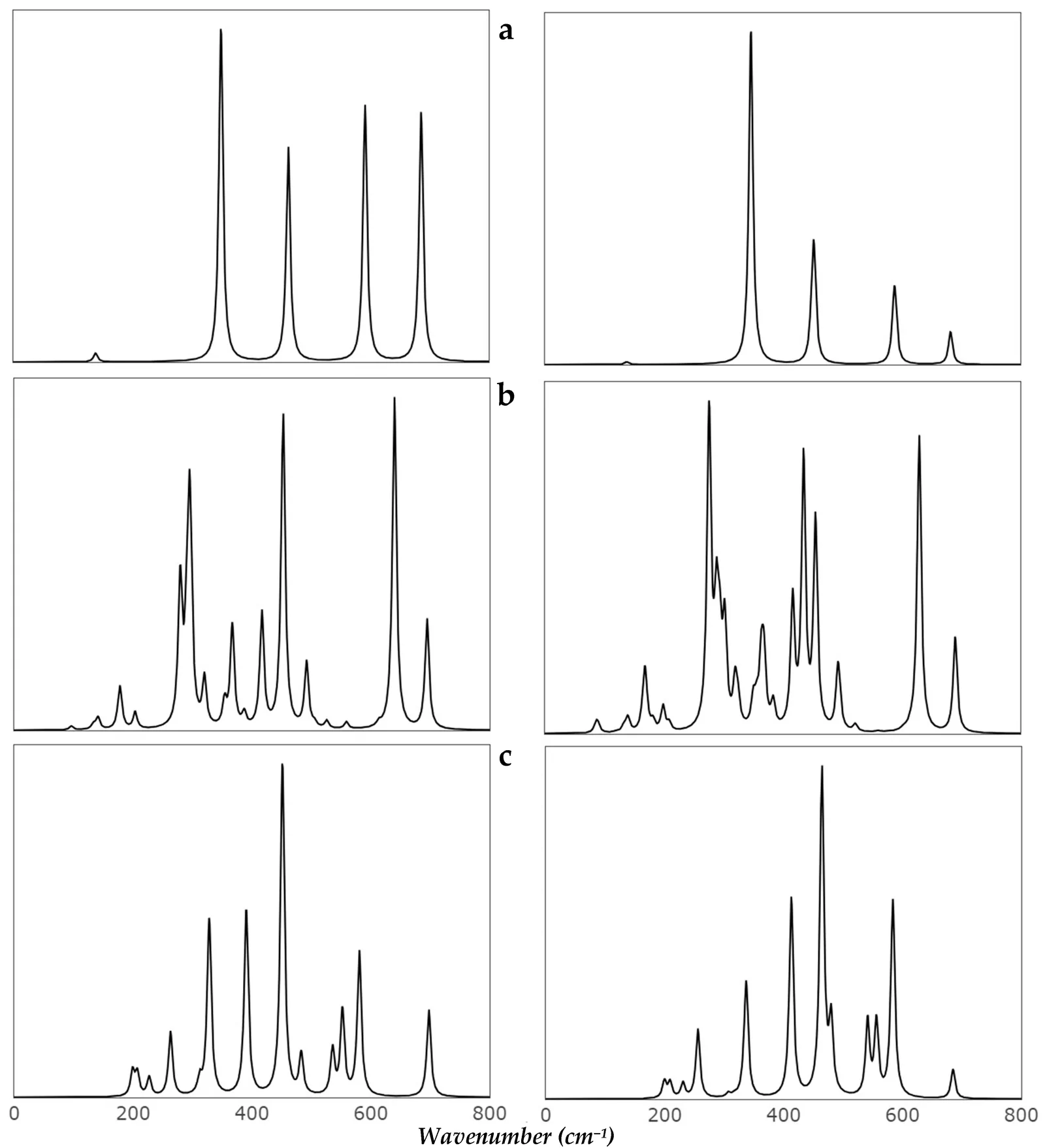
B3LYP-simulated Raman spectra of CdFO across various degrees of inversion (X = 0.0 (**a**), 0.5 (**b**), 1.0 (**c**)) and two different magnetic configurations (FM and AFM), arranged from left to right for FM and AFM configurations.

In CdFO, the five Raman-active modes are confined to the range of approximately 100 cm^−1^ to 700 cm^−1^ at X = 0.0, as shown in Figure 6. These frequencies are consistent with typical Raman vibrational frequencies observed in spinel ferrites. The modes observed start from the lower frequency end with (T_2g_) at 138.26 cm^−1^, which is barely visible and of very low intensity, similar to previous observations in related ferrite systems. The second mode, doubly degenerate (E_g_) at 349.37 cm^−1^, exhibits the highest intensity compared to the other two triply degenerate modes (462.57 cm^−1^ and 591.82 cm^−1^) and the non-degenerate mode (A_1g_) at 686.15 cm^−1^, which show almost equal but slightly lower intensities.

The analysis of the vibrational modes assigns the 138.26 cm^−1^ peak to bending motions involving O-A-O and A-O-B linkages. The 349.37 cm^−1^ peak involves more complex motions, including the stretching of atoms in octahedral coordination (B-O-B, O-B-O, and A-O-B) and bending of O-A-O and A-O-B, likely contributing to its high observed intensity. The peak at 462.57 cm^−1^ is attributed to exclusive stretching motions of O-A-O and A-O-B, while the 591.82 cm^−1^ peak encompasses all possible bending motions between cations and anions. The highest frequency peak at 686.15 cm^−1^ is associated with stretching modes of A−O and bending modes of A−O−B.

Despite minor differences in calculated energy between the FM and AFM structures, changes in spin arrangement lead to significant variations in the relative intensities and minor shifts in peak positions. For instance, in FM, the three higher frequency peaks, which had almost equal intensities, show a gradual decrease in their intensities, with the E_g_ peak becoming stronger. The A_1g_ peak becomes very weak, with a significant drop in its intensity. This behavior contrasts with its role in Zn, Mg, and Co ferrites, where it stands as the highest peak for both FM and AFM configurations, as reported in previous studies [27,33,34,35]. Additionally, there are small shifts in peak positions, approximately 2–4 cm^−1^, except for a significant shift of about 10 cm^−1^ at 463 cm^−1^.

In mixed-spinel structures, symmetry breaking introduces new active peaks, resulting in more complex spectral features. In FM, small twin appears at 133 cm^−1^, 140 cm^−1^, and stronger peaks at 177 cm^−1^ and 203 cm^−1^, followed by two high-intensity peaks at 279 cm^−1^ and 294 cm^−1^, and a smaller one at 319 cm^−1^. The E_g_ peak drops in intensity and appears at 354 cm^−1^, accompanied by a moderate new peak at 367 cm^−1^ followed by a small active peak at 387 cm^−1^. The second T_2g_ peak (462.5 cm^−1^) in the normal structure splits into two strong peaks at 417 cm^−1^ and 452 cm^−1^, followed by a moderate new peak at 491 cm^−1^. The third T_2g_ peak (591.8 cm^−1^) shows a significant increase in intensity and shifts to 613 cm^−1^. Finally, the A_1g_ peak (686 cm^−1^) in the normal structure shifts to 694 cm^−1^. In the AFM phase, the spectra display the same newly activated peaks with changes in relative intensity that distinguish the AFM from FM spectra.

In the inverse-order spectra, dramatic changes are observed compared to the normal and mixed configurations. In the FM phase, beginning our analysis from the lower frequency region, no features are observed below approximately 200 cm^−1^. The first new twin small peaks appear at 199 cm^−1^ and 207 cm^−1^, followed by another small peak at 227 cm^−1^ and a moderate one at 263 cm^−1^. Beyond 300 cm^−1^, stronger peaks start to emerge, beginning at 328 cm^−1^ with a small shoulder at 312 cm^−1^, then another strong peak at 391 cm^−1^, which corresponds to the 463 cm^−1^ peak observed in the normal phase. The most intense new peak is noted at 451 cm^−1^, accompanied by a new small peak at 483 cm^−1^. Towards the end of the spectrum, the peak at 592 cm^−1^ in the normal phase splits into three clearly separate peaks at 536 cm^−1^, 552 cm^−1^, and 581 cm^−1^, each growing gradually in intensity. The A_1g_ peak at 698 cm^−1^ shows a drop in intensity compared with its counterpart in the normal phase. Table 6 lists the frequencies of the AFM spectra and compares them with the corresponding FM counterpart for both X = 0.0 and X = 1.0, noting that most new activated peaks in FM are detected in AFM with shifts in positions, reaching up to approximately 30 cm^−1^ in the middle region.

The comparison between the simulated Raman frequencies for normal CdFO and those reported in previous experimental studies, as listed in Table 7, reveals some variations and potential inconsistencies in mode assignments. For example, frequencies around 314 cm^−1^ [24] and 331 cm^−1^ [25] have been attributed to different Raman modes (E_g_ and T_2g_) across various studies. Such variability in assignments may reflect challenges in experimental characterization, such as overlapping peaks, instrumental resolution limitations, or the influence of structural distortions and defects, which can complicate precise mode identification.

In the present study, the calculated frequencies provide a consistent assignment within the framework of the normal spinel structure. These assignments align with the symmetry properties expected for a normal spinel structure and are derived under idealized conditions using the B3LYP functional. While the calculated values are systematically higher than experimental ones, likely due to the exclusion of temperature effects and structural defects, they offer a consistent reference point for interpreting vibrational modes.

One interesting observation is the absence of a peak near 500 cm^−1^ in the experimental spectra [23,24,25] This could potentially be explained by the low intensity of this peak in the AFM configuration observed in the current calculations. Since the peak at approximately 500 cm^−1^ exhibits higher intensity in the FM configuration, its absence in experimental spectra may indirectly support the presence of the AFM configuration in the experimental sample, where such low-intensity peaks might not be detected. Additionally, the ~500 cm^−1^ peak may overlap with nearby peaks, complicating its identification in experimental spectra. This highlights the interplay between magnetic ordering and spectral features, underscoring the challenges in assigning peaks in this region.

The A_1g_ mode provides another example where experimental assignments show variation, reported in the range of 634–674 cm^−1^ in earlier studies [23,24,25]. In the current calculations, this mode is assigned to 686 cm^−1^ (FM) and 682 cm^−1^ (AFM). These calculated values are slightly higher but remain within reasonable proximity, suggesting that theoretical methods can complement experimental results to refine mode assignments.

## 3. Materials and Methods

This research employs the principles of density functional theory (DFT), using the CRYSTAL_17_ software [37]. The functionals utilized in these simulations include the global hybrid functional B3LYP [38], which integrates 20% Hartree–Fock exchange, PBE0 with a 25% incorporation of Hartree–Fock exchange [39], and the distance-dependent HSE06 functional [40], which adjusts the fraction of Hartree–Fock exchange based on electron separation. These choices help mitigate the typical self-interaction inaccuracies found in straightforward DFT functionals. Although DFT + U is a widely used approach for describing transition-metal oxides, we employed hybrid functionals in this work due to their improved ability to describe both electronic structure and vibrational properties without relying on system-specific Hubbard U parameters [41]. This avoids the need for empirical tuning and enables a more transferable description of the exchange–correlation effects. Gaussian-type orbitals form the basis set for all elements analyzed [42,43,44].

The precision of our computational approach is maintained through stringent convergence criteria for the Coulomb and exchange series, defined by five truncation thresholds, with the first four (T_1_–T_4_) set at a value of 8 and the fifth (T_5_) at 16. The energy convergence thresholds for self-consistent field (SCF) calculations are 10^−8^ and 10^−10^ Hartree for structural optimizations and vibrational frequency assessments, respectively. The optimization routine involved a full adjustment of lattice constants and atomic positions, aiming for a root mean square displacement of less than 10^−5^ Å. The Monkhorst–Pack grid [45,46] for k-point sampling was finely adjusted with shrinking factors, providing 29 distinct points within the first Brillouin zone for ZFO. These computations encompass the evaluation of atomic configurations, total energy calculations, and the analysis of electronic and magnetic properties across varying degrees of inversion in CdFO. The IR and Raman spectra were deduced from the equilibrium configurations, highlighting both the frequency and intensity of vibrationally active modes. Additionally, spin-polarized studies were conducted to assess the effect of magnetic order on the material’s electronic and structural characteristics. In scenarios where the system exhibits ferromagnetism, magnetic moments are aligned uniformly, whereas in antiferromagnetic settings, these moments are oriented in opposition between pairs of Fe atoms within the structure.

Frequency calculations (wavenumber ωp) are derived from the secondary energy derivatives relative to atomic displacements at the Γ point [47,48]. These calculations consider the mass and Cartesian coordinates of the displaced atoms, allowing for straightforward simulation of isotopic effects on vibrational frequencies through mass adjustments in the relevant equations. The intensity of IR absorption for each vibrational mode is quantified using a CPHF/KS method [49,50,51], and Raman intensities are determined analytically using solutions from first and second-order coupled Perturbed–Hartree–Fock/Kohn–Sham equation [52,53].

## 4. Conclusions

In this study, we employed different hybrid functionals, including B3LYP, HSE06, and PBE0, to investigate the relative stabilities of Cd ferrite geometries under varying spin arrangements and inversion degrees. Our calculations identified the normal spinel phase as the most stable configuration, with energy differences between different spin arrangements within this phase (FM, FRI, and AFM) found to be minimal, ranging from ~0.005 to 0.008 eV. When zero-point energy was included, the differences increased slightly to ~0.023 eV, underscoring the subtle impact of spin ordering on the stability of the normal spinel phase. The simulated Raman spectra aligned qualitatively with experimental trends, though some shifts in frequency were observed. The absence of a detectable peak near ~500 cm^−1^ in experimental spectra could be attributed to its low intensity in the AFM configuration and potential overlap with nearby peaks. This observation supports the presence of AFM-like ordering in the experimental sample. Furthermore, the Raman-active modes (T_2g_, E_g_, and A_1g_) were consistently assigned based on the theoretical framework, with calculated frequencies providing a valuable reference for refining experimental mode assignments.

While our results provide important insight into the structural, vibrational, and magnetic properties of Cd ferrite, we acknowledge that further experimental validation—particularly using polarization-resolved Raman spectroscopy and magnetic characterization—is essential to confirm the predicted trends and assess the role of spin ordering with greater certainty. Overall, this study demonstrates the strength of combining hybrid-functional DFT with vibrational analysis to uncover subtle spin–lattice interactions. The use of multiple hybrid functionals enabled a robust evaluation of energetic trends, while the Raman simulations provided new perspectives on the interplay between magnetic configurations and vibrational dynamics. These findings lay a foundation for future experimental and theoretical investigations of Cd ferrite and related spinel oxides.

## Figures and Tables

**Table 1:** Total energies (in Hartree) for CdFO spinel geometries (Cd_1-X_Fe_X_)[Cd_X_Fe_2-X_O_4_] at X = 0, 0.5, and 1, calculated with various functionals. *E*_R_ refers to energies relative to the AFM normal spinel structure (In eV).

Functionals		(X = 0)			(X = 0.5)			(X = 1)		
		FM	FRI	AFM	FM	FRI	AFM	FM	FRI	AFM
B3LYP	*E*	−16,591.3505	−16,591.3506	−16,591.3508	−16,591.3068	−16,591.3192	−16,591.3150	−16,591.2923	−16,591.3055	−16,591.3173
	*E*_R_	(0.008)	(0.005)	(0.000)	(1.197)	(0.859)	(0.974)	(1.591)	(1.232)	(0.911)
HSE06	*E*	−16,590.0006	−16,590.0008	−16,590.0009	−16,589.95282	−16,589.9644	−16,589.9602	−16,589.9379	−16,589.9502	−16,589.9613
	*E*_R_	(0.008)	(0.003)	(0.000)	(1.308)	(0.993)	(1.107)	(1.714)	(1.379)	(1.077)
PBE0	*E*	−16,590.0070	−16,590.0071	−16,590.0072	−16,589.9591	−16,589.9702	−16,589.9660	−16,589.9438	−16,589.9558	−16,589.9665
	*E*_R_	(0.005)	(0.003)	(0.000)	(1.308)	(1.006)	(1.121)	(1.725)	(1.398)	(1.107)

**Table 2:** Thermodynamic properties (eV/cell) at T = 298.15 K and *p* = 0.101325 MPa for various CdFO configurations, including electronic energy per bell (*E*_L_), zero-point energy (*E*_0_), thermal contribution to vibrational energy (*E*_T_), and entropy (S), calculated at the B3LYP level. Parentheses show energy relative (in eV) to the ground state (AFM, X = 0.0).

Energy	X = 0		X = 0.5		X = 1	
	FM	AFM	FM	AFM	FM	AFM
*E*_L_	−451,473.6017	−451,473.6077	−451,472.4116	−451,472.6339	−451,472.0165	−451,472.6978
	(0.006)	(0.000)	(1.196)	(0.973)	(1.591)	(0.909)
*E*_0_	0.848033	0.837199	0.838940	0.832946	0.829726	0.835760
*E*_T_	0.419251	0.424238	0.424223	0.4273647	0.424812	0.423246
S (meV/(cell.K))	2.413010	2.451891	2.474677	2.5096526	2.458753	2.469079
PV	0.000108	0.000108	0.000109	0.0001084	0.000108	0.000106
TS	0.719439	0.7310314	0.737824	0.7482529	0.733077	0.736156
*E*_T_ + PV-TS	−0.300078	−0.3066847	−0.313492	−0.3207797	−0.308156	−0.312802
*E*_L_ + *E*_0_ + *E*_T_ + PV-TS	−451,473.0537	−451,473.0772	−451,471.8861	−451,472.1217	−451,471.4950	−451,472.1748
	(0.023)	(0.000)	(1.191)	(0.955)	(1.582)	(0.902)

**Table 3:** Magnetic moments (μB) for different magnetic configurations (FM, FRI, and AFM) in CdFO geometries (Cd_1-X_Fe_X_) [Fe_2-X_O_4_], calculated using various functionals.

Functionals	CdFO		Fe (O_h_) μB	Fe (T_d_) μB	Cd (O_h_) μB	Cd (T_d_) μB	O μB	
B3LYP	X = 0	FM	4.317 (4)			0.063 (2)	0.326 (8)	
		FRI	4.309 (3)	_	_	0.032 (2)	0.313 (2)	
			−4.295				0.113 (6)	
		AFM	4.301 (2)	_	_	0.000 (2)	−0.102 (4)	
			−4.301 (2)				−0.102 (4)	
	X = 0.5		4.304 (3)	4.275	0.039	0.074	0.357	0.368
		FM					0.341(3)	0.318(3)
			4.278(3)	−4.214	−0.008	0.074	0.111	0.364
		FRI					−0.028 (3)	0.308 (3)
		AFM	4.274 (2) −4.287	−4.234	−0.019	0.023	−0.041	0.120
							−0.164 (2)	−0.010 (2)
							−0.055	0.291
	X = 1	FM	4.341 (2)	4.261 (2)	0.045 (2)	–	0.350 (4)	0.326 (4)
		FRI	4.304 (2)	−4.194	−0.002 (2)	_	0.054 (2)	0.341 (2)
				4.256			−0.042 (2)	0.315 (2)
		AFM	4.270 (2)	−4.195 (2)	−0.053 (2)	–	0.047(4)	−0.057(4)
HSE06	X = 0	FM	4.372 (4)	–	–	0.064 (2)	0.298 (8)	
		FRI	4.365 (3)	_	_	0.033 (2)	0.287 (2)	
			−4.354				0.104 (6)	
		AFM	4.359 (2)	_	_	0.000 (2)	−0.093 (4)	
			−4.360 (2)				0.094 (4)	
	X = 0.5	FM	4.361 (3)	4.337	0.035	0.075	0.324	0.334
							0.314 (3)	0.290 (3)
		FRI	4.337 (3)	−4.279	−0.009	0.075	0.099	0.333
							−0.025 (3)	0.282 (3)
		AFM	4.334 (2)−4.347	−4.298	−0.018	0.024	−0.038	0.110
							−0.154 (2)	−0.006 (2)
							−0.045	0.267
	X = 1	FM	4.400 (2)	4.318 (2)	0.043 (2)	-	0.319 (4)	
							0.319 (4)	
		FRI	4.366 (2)	−4.257	−0.005 (2)	-	0.051 (2)	0.312 (2)
				4.312			−0.044 (2)	0.292 (2)
		AFM	4.334 (2)	−4.257 (2)	−0.055 (2)	-	0.044 (4)	
							−0.055 (4)	
PBE0	X = 0	FM	4.380 (4)	–	–	0.064 (2)	0.294 (6)	
		FRI	4.374 (3)	_	_	0.033 (2)	0.283 (2)	
			−4.364				0.102 (6)	
		AFM	4.369 (2)	_	_	−0.000 (2)	−0.093 (4)	
			−4.369 (2)				0.093 (4)	
	X = 0.5	FM	4.369 (3)	4.346	0.035	0.075	0.319	0.331
							0.310 (3)	0.286 (3)
		FRI	4.346 (3)	−4.289	0.075	−0.010	0.097	0.329
							−0.026 (3)	0.279 (3)
		AFM	4.344 (2)−4.356	−4.308	−0.018	0.024	−0.038	0.109
							−0.153 (2)	−0.007 (2)
							−0.046	0.266
	X = 1	FM	4.409 (2)	4.327 (2)	0.043 (2)	-	0.315 (4)	
							0.296 (4)	
		FRI	4.376 (2)	−4.268	−0.005 (2)	-	0.050 (2)	0.308 (2)
				4.321			−0.045 (2)	0.288 (2)
		AFM	4.346 (2)	−4.268 (2)	−0.055 (2)	-	0.043 (4)	
							−0.055 (4)	

**Table 4:** Spin-resolved electronic band gaps in CdFO for majority (α) and minority (β) spin channels, calculated using B3LYP, HSE06 and PBE0 functionals (In eV).

Primitive Formula	Inversion Degree	Spin Arrangement	Band Gap					
			B3LYP		HSE06		PBE0	
			$E_{g}^{\;\alpha }$	$E_{g}^{\;\beta }$	$E_{g}^{\;\alpha }$	$E_{g}^{\;\beta }$	$E_{g}^{\;\alpha }$	$E_{g}^{\;\beta }$
(Cd_2_)[Fe_4_]O_8_	X = 0.0	FM	4.884	3.012	4.719	3.120	5.436	3.790
		FRI	3.430	2.916	3.542	3.043	4.311	3.763
		AFM	3.112	3.112	3.251	3.246	3.977	3.977
(CdFe)[CdFe_3_]O_8_	X = 0.5	FM	4.951	3.018	4.740	2.950	5.455	3.633
		FRI	3.221	3.120	3.228	3.161	3.988	3.876
		AFM	3.111	3.145	3.144	3.260	3.870	4.001
(Fe_2_)[ Cd_2_Fe_2_]O_8_	X = 1.0	FM	5.078	2.677	4.858	2.700	5.561	3.392
		FRI	3.430	2.916	3.750	2.922	4.502	3.643
		AFM	3.523	3.005	3.565	3.079	4.305	3.808

**Table 5:** Comparison of previously reported and currently calculated IR mode frequencies for normal CdFO.

Vibrational Modes (cm^−1^)	Ref. [31]	Ref. [32]	Ref. [23]	Current Work (X = 0.0)	
				FM	AFM
v_1_	549.61–553.47	553–578	548–549	536.36	527.47
v_2_	424.26–410.75	438–403	374–380	350.10	348.04
v_3_				279.02	285.00 Sh-305.12
v_4_				153.38	137.49

**Table 6:** Frequency comparison of vibrational modes (In cm^−1^) between inverse and normal cubic structures of CdFO within the FM spin order.

	X= 0.0	X =1.0	
	FM	FM	AFM
**T_2g_ (1)**	138.26	I	I
		199.10	199.57
		206.96	209.02
		227.00	230.68
		262.98	255.89
		302.33	
		I	306.95
			317.03
		312.22	
		324.24	332.23
		328.27	
**E_g_ (1)**	349.37	I	337.12
**T_2g_ (2)**	462.57	390.50	413.13
			464.58
		451.39	479.93
		483.01	I
**T_2g_ (3)**	591.82	535.77	541.58
		552.35	556.23
		580.72	583.89
**A_1g_**	686.15	697.72	685.11

**Table 7:** Comparison of previously reported and currently calculated Raman mode frequencies (In cm^−1^) for normal CdFO.

Raman Symmetry Assignment	Ref. [36]	Ref. [23]	Ref. [24]	Ref. [25]	Current Work (X = 0)	
					FM	AFM
T_2g_ (1)					138	137
E_g_		325–329		314	349	347
T_2g_ (2)		438–445	331		463	452
T_2g_ (3)			446	472	592	588
A_1g_	644	634–636	652	674	686	682

## Data Availability

The original contributions presented in this study are included in the article. Further inquiries can be directed to the corresponding author.
